# Genome–wide microRNA expression profiling in placentas from pregnant women exposed to BPA

**DOI:** 10.1186/s12920-015-0131-z

**Published:** 2015-09-07

**Authors:** Bruna De Felice, Francesco Manfellotto, Annarita Palumbo, Jacopo Troisi, Fulvio Zullo, Costantino Di Carlo, Attilio Di Spiezio Sardo, Noè De Stefano, Umberto Ferbo, Marco Guida, Maurizio Guida

**Affiliations:** DISTABIF–Department of Environmental, Biological and Pharmaceutical Sciences and Technologies, University of Naples II, Via Vivaldi 43, 81100 Caserta, Italy; Department of Gynecology and Obstetrics, University of Salerno, Salerno, Italy; Department of Gynecology and Obstetrics, University of Naples “Federico II”, Naples, Italy; Department of Pathology, S.Giuseppe Moscati Hosptal, Avellino, Italy; Department of Biological Sciences, University of Naples, Naples, Italy

## Abstract

**Background:**

Bisphenol A (BPA) is an environmental compounds is known to possess endocrine disruption potentials. Bisphenol A has epigenetic effects as deregulated expression of microRNAs; such epigenetic marks can induce up/down alterations in gene expression that may persist throughout a lifetime. Bisphenol A (BPA) exposure has been documented in pregnant women, but consequences for development of offspring after BPA exposure during pregnancy are not yet widely studied. Therefore, the aim of this study was to gain a comprehensive understanding of microRNAs changes in the placenta transcriptome from pregnant women subjected to therapeutic abortion for fetal malformation and correlate the impact of gestational exposure to BPA on these developmental changes.

**Methods:**

We performed a comparative analysis of genome wide miRNA expression in placentas from pregnant women exposed to BPA using microarray technology to identify miRNAs which were aberrantly expressed in placentas from malformed fetuses. The expression changes of differential expressed miRNAs in the samples used for microarray were confirmed by qPCR . Beside, we applied various bioinformatics tools to predict the target genes of the identified miR-146a and explore their biological function and downstream pathways.

**Results:**

We found that miR-146a was significant overexpressed and correlated significantly with BPA accumulation in the placenta from pregnant women living in a polluted area and undergoing therapeutic abortion due to fetal malformations. Beside, we applied various bioinformatics tools to predict the target genes of miR-146a and explore their biological function and downstream pathways.

**Conclusions:**

For the first time, we found, in humans, that miR-146a was significant over-expressed and correlated significantly with BPA accumulation in the placenta. Our results lead to the suggestion that miRNAs could be potential biomarkers to clarify the mechanisms of environmental diseases.

## Background

Bisphenol A is produced in high volume worldwide for use in a variety of industrial and consumer products. Exposure to BPA at concentrations detected in humans has been reported to affect neurological, cardiovascular and metabolic diseases (such as diabetes), and even cancers [1]. Low level BPA exposure may impact placental tissue development and function in humans. For its toxicity and estrogenic activity, BPA is a potential concern to fetal and infant health endpoints since has been found in a variety of human tissues and body fluids as follicular and amniotic fluid [2], umbilical cord blood and placental tissue [3], and breast milk [4].

Recent data from in vitro studies suggest that fetal exposure to BPA can occur throughout placental exchange [5] and that BPA exposure at doses (0.02 - 0.1 μg/mL) close to those found in some pregnant women can induce cell death in isolated human cytotrophoblast cells [6].

It is known that prenatal exposure to BPA has irreversible effects on prenatal reproductive development [7, 8]; and was associated with a significant increase in the birth weight of male infants [7]. Beside, effects on anthropometric measures at 4 years of age [9] adipokine levels in 9-year-old children [10], child wheeze [11] and child behaviour [12], and obesity in school-age children. The perinatal period corresponds to the temporal window when the enamel of the permanent incisors and first molars is being formed. BPA may be involved in molar incisor hypo-mineralization (MIH) by having an adverse effect on amelogenesis [2].

BPA, as endocrine disruptor may be one of the many critical factors in aberrant development of external genitalia that manifests as hypospadias [13].

Ambient air pollution is associated with birth weight and preterm birth and health outcomes of maternal BPA exposure are fetal toxicity, impairing fetal growth and associated with prenatal and postnatal side effects [14, 15]. In mouse prenatal exposure to bisphenol A affects adult murine neocortical development and thalamocortical connections [16] and disrupts the normal function of the reproductive axis in prepuberal male rats [17], The effects of the BPA may be exerted at different levels of the axis and may be dependent on the dose, manner of administration, and the moment of exposure to the disruptor.

Prenatal exposure to low doses of bisphenol A (BPA) may cause epigenetic changes as histone modifications, DNA hypermethylation/hypomethylation at CpG islands near gene promoter regions, and deregulated expression of non-coding RNAs, including microRNAs. These epigenetic marks can induce up/down alterations in gene expression that may persist throughout a lifetime resulting in adverse health effects such as infertility, neural and immune disorders, and late-onset complex diseases as cancers and diabetes.

MicroRNAs (miRNAs) are approximately 22 nucleotide long non-coding RNAs capable of regulating gene expression post-transcriptionally through imperfect complementarity with a target mRNA, and are considered a mode of epigenetic regulation [18].

Aberrant miRNA expression has been implicated in a several cellular processes and pathogenic pathways of a number of diseases [19, 20]. Evidence is rapidly growing that miRNA regulation of gene expression may be affected by environmental chemicals [21, 22]. The accumulating evidence linking miRNAs to environmental chemicals, coupled with the unique regulatory role of miRNAs in gene expression, makes microRNAs potential biomarkers for better understanding the mechanisms of environmental diseases.

The exact mechanisms by which the environmental factors alter miRNAs expression are not completely understood. A conceptual model that may help explain the mechanism underlying miRNAs dysregulation induced by environmental chemicals is centred on ‘inflammation and oxidative stress, having a direct impact on miRNAs deregulation. However, this model may be limited to those environmental chemicals for which inflammation and oxidative stress are primary mediators of toxicity.

Therefore, it has been assumed that chemicals in the environment might cause miRNAs deregulation by increasing the oxidative stress and / or the activation of inflammatory responses. Indeed, both processes are implicated in various diseases [21].

Regarding miRNAs, it has been shown that BPA exposure of human placental cell lines can alter miRNA expression levels, and in particular, hsa-miR-146a was strongly induced by BPA treatment. This resulted in both slower proliferation rate and higher sensitivity to the DNA damaging agent bleomycin [23]. TM4 mouse sertoli cell line exposed to BPA for 24 h was reported to have two-fold up or down-regulated 37 miRNAs, and most of miRNAs were down-regulated over the course of BPA treatment [24]. Placenta is able to accumulate BPA, as well as transfer BPA to the fetus, which has little capacity to deactivate BPA. Since placenta is generally discarded after delivery, such tissue is ideal to use for biomonitoring of potential toxicants. Placental delivery takes place after the birth of the child, so it is a non-invasive, long-term sample that can be analyzed for the passage of toxicants into the fetus [25].

In the present study, we performed a comparative analysis of genome wide miRNA expression in placentas from pregnant women exposed to BPA using microarray technology to identify miRNAs that were aberrantly expressed in placentas from malformed fetuses. We found that miR-146a was significant over-expressed and correlated to BPA accumulation in the placenta as a measure of fetal exposure related to fetal malformations. In addition, we applied various bioinformatics tools to predict the target genes of the identified miR-146a and explore their biological function and downstream pathways.

## Results

Forty placenta samples from pregnant women living in a polluted area (patient group subjected to therapeutic abortion occurring in the second trimester of pregnancy for fetal malformation) and 40 placenta samples from pregnant women living in a non-polluted area (control group: women with a healthy pregnancy) were used for genome-wide miRNA expression profiling.

### Subject characteristics and exposure

The characteristics of the 80 study subjects are summarized in Table 1. Maternal age, gestational age, body mass index, and gender ratio of the fetuses did not differ between malformed and normal fetuses (*P* > 0.05 for all, Table 1).

**Table 1:** Biological characteristics of the study population

Features	Total number (%)
Healthy pregnant women with normal fetus	40 (50)
Healthy pregnant women iving in a polluted area; these	40 (50)
women were subject to therapeutic abortion	
Age	
25-34	40 (50)
35-40	40 (50)
Week of gestation	
13-16	7 (17,5)
17-20	10 (25)
21-24	12 (30)
>24	11 (27,5)
Maternal BMI	27.5 (Mean)
Gender ratio	
Male	42 (52.5)
Female	38 (47.5)
Malformation	
Trisomy 21	6 (20)
Nervous system diseases	13 (26,7)
Other	5 (16,6)
Cardiac malformation	14 (30)
Hydrops fetalis	2 (6,7)

### Microarray analysis

In this study, 1349 miRNAs including 1205 human miRNAs and 144 human viral miRNAs were profiled. We dentified differentially expressed miRNAs in placenta samples according to the presence or absence of malformed fetuses. Thirty-four miRNAs had at least a 2.5-fold difference in expression at *P* < 0.05/ Twelve miRNAs were up-regulated and six miRNAs were down-regulated in placentas samples from malformed fetuses (Table 2). The expression changes of differential expressed miRNAs in the samples used for microarray were confirmed by qPCR. Specifically, miR-146a was strongly induced.

**Table 2:** List of significantly dysregulated miRNAs from placentas after BPA exposure

miRNA	Fold Change	*P* Value
Up-regulated		
hsa-mir-1243	2.721	.039
hsa-mir-519e-3p	3.768	.042
hsa-mir-371-3p	3.183	.033
hsa-mirplus-c1066	4.163	.047
hsa-mir-146a	8.443	.027
hsa-mir-29a	2.067	.046
hsa-mir-1256	4.618	.037
hsa-miR-21	3.22	.087
hsa-miR-26b	3.43	.067
hsa-miR-29a	3.21	.029
hsa-miR-335	4.31	.047
hsa-miR-376c	2.74	.010
Down-regulated		
hsa-mir-605	−5.662	.034
hsa-mir-571	−5.079	.024
hsa-mir-23b-5p	−4.681	.011
hsa-mir-885-5p	−4.304	.024
hsa-mir-1471	−4.276	.040
hsa-let-7a-2-3p	−3.649	.047

### Differences in miR-146a expression between patients and control group

**Fig. 1:**
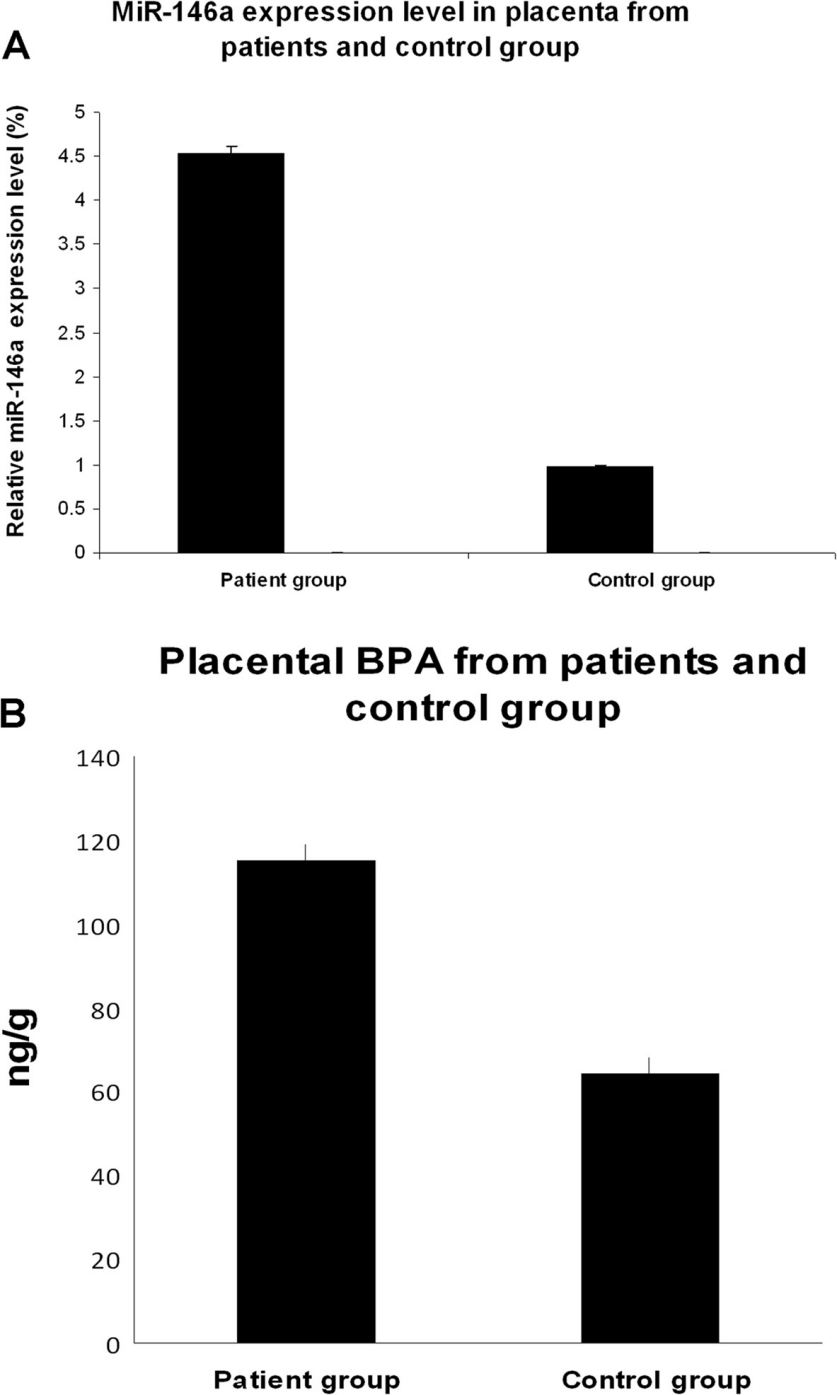
MiR-146a expression levels and bisphenol A (BPA) value in placenta from pregnant women living in polluted area and pregnant women living in a non-polluted area. **a** The expression of mir-146a was studied in placenta from 40 pregnant women, undergoing therapeutic abortion occurring in the second trimester of pregnancy for fetal malformations and from 40 pregnant women living in a non-polluted area by microRNA assay-based quantitative RT-PCR following the Delta-Delta Ct method. RNU6B was utilized for an endogenous reference to standardize microRNA expression levels. The results were expressed as relative expression levels after calibration with the universal reference data. The asterisk indicates a significant difference between patients with control group. *P* < 0.05. **b** Placental bisphenol A (BPA) value between 40 pregnant women, undergoing therapeutic abortion occurring in the second trimester of pregnancy for fetal malformations and from 40 pregnant women living in a non-polluted area *P* < 0.05

miR-146a expression was significantly increased in placenta samples taken from pregnant women whose fetuses had malformations compared with women with a healthy pregnancy (*p* < 0.05; Fig. 1a).

### BPA analysis

**Fig. 2:**
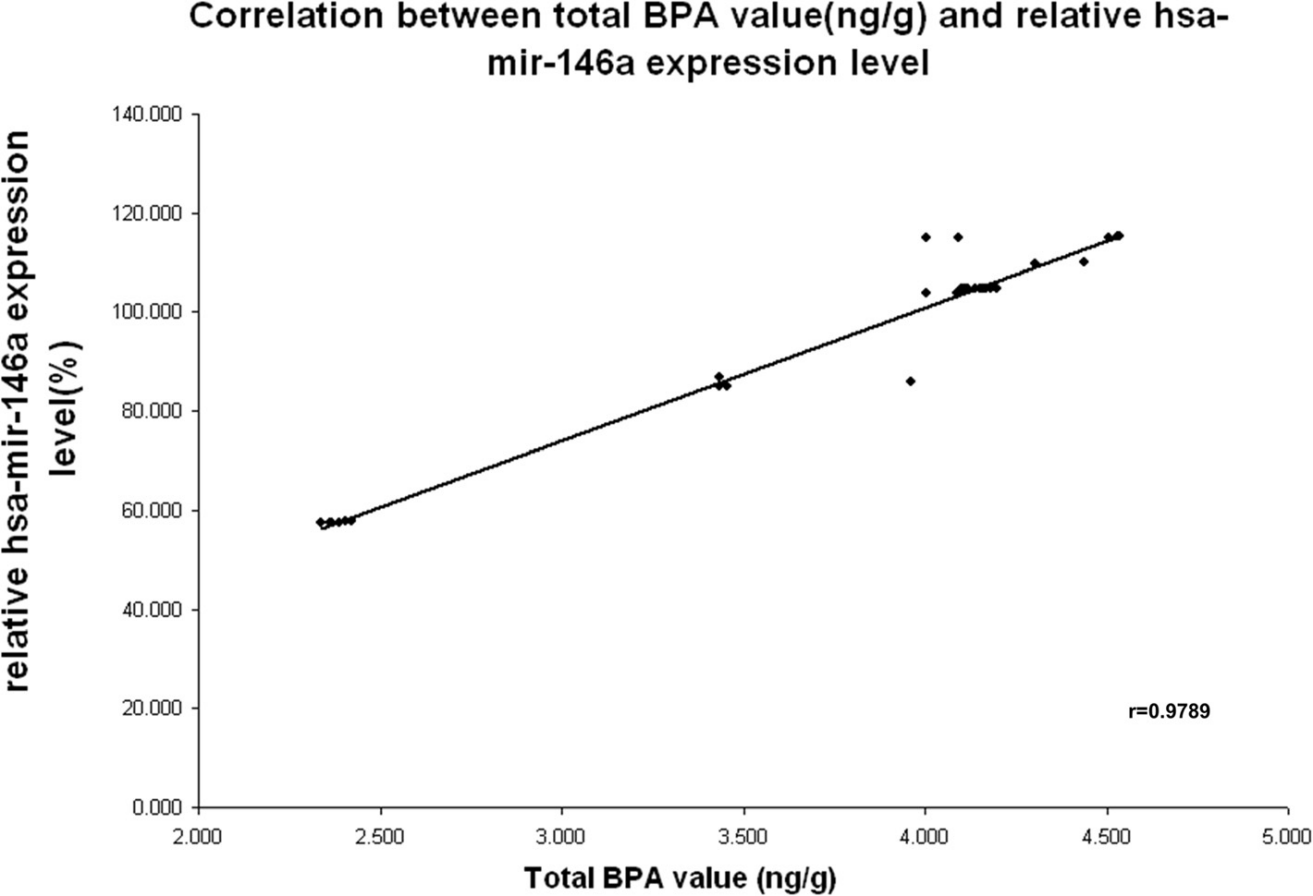
Correlation between total BPA level and relative has-miR-146a expression level in placenta from pregnant women living in polluted area. A positive correlation between miR-146a and BPA expression levels was observed (Pearson’s correlation coefficient = 0.9789, *P* = 0.003)

The BPA exposure has been studied on a subset of the enrolled women (40 patients and 40 controls). BPA was significant absent in the control group, while were detected only in the patients subjected to therapeutically abortion (Fig. 1b). High level of BPA was associated with a significant increased miR-146a expression. Figure 2 shows the positive correlation between BPA level and miR-146a relative expression in placentas from pregnant women living in polluted area (r = 0. 9789) (*p* < 0.05).

### Target mapping and functional analysis: potential interactions of miR-146a

To explore the functional significance of the miRNAs investigated, we applied KEGG (www.genome.jp/kegg/), a pathway analysis database, to the target genes identified for miR-146a using miRanda and TargetScan. The enriched pathways we identified appeared to be largely overlapping, since many target genes were in more than one pathway. Among the top-ranked pathways, we found those related to general functions (e.g., enzymes, cell cycle) and others with more specific functions, including a high proportion of pathways related to signal transduction, transcription factors, cancer and nervous system-related genes.

Identifying targets can facilitate elucidation of the role of miR-146a in BPA exposure. Usually, one miRNA has hundreds of target genes, and a group of miRNAs co-modulated a signalling pathway. MiR-146a target genes were obtained from the databases mentioned above. Subsequently, these target genes were mapped to KEGG, to identify molecular function, signalling pathways and their cell location.

**Fig. 3:**
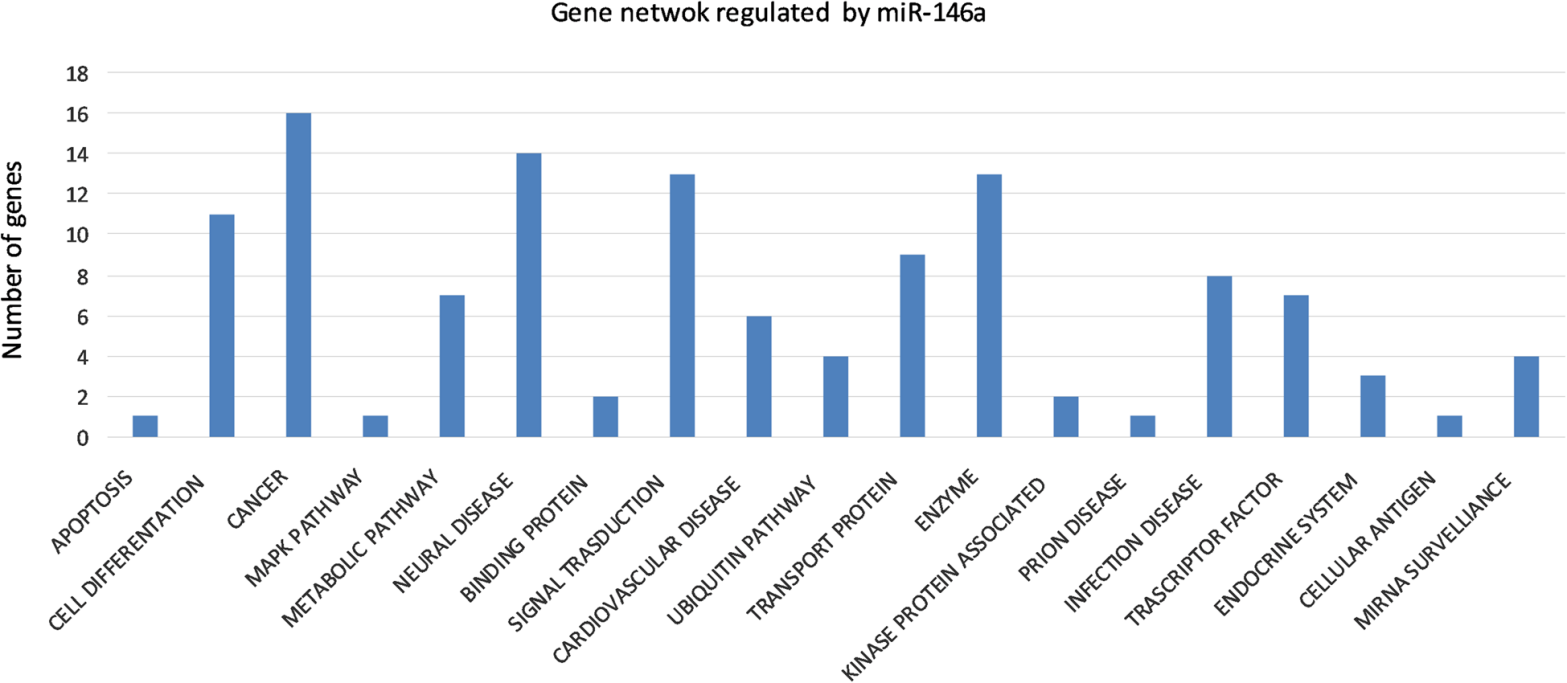
Gene networks regulated by miR-146a. Predicted miR-146a target genes identified by TargetScan and Miranda

There were 19 significant (*p* < 0.01) biological functions associated with miR146a target genes under BPA exposure (Fig. 3). One of these functions described neural disease genes pathway, which was the second most significant function on the list (*p* = 0.002) and comprised 12 genes: IRAK1, MYT1, ROBO1, LRRTM2, GRID1, SORT1, BCL11A, SYT1, NPAS4,MLL2, DNAL1, EDNRB. Such genes were involved in differentiation and proliferation of sympathetic neuron, communication disorders, import of L-glutamic acid, outgrowth of sensory axons, brain-derived neurotrophic factor, damage of hippocampal cells.

Endocrine system genes pathway included genes as TP53 regulating kinase and MED1, Mediator complex subunit 1, which have been shown may interact with Androgen receptor, Estrogen receptor alpha, and p53.

Another biological function describrd cardiovascular disease gene as ABL2, ABL2 and EDNRB. Beside, target genes of differential expression miR-146a were enriched in several cancer-related pathways, including the ErbB, p53, Toll and mTOR-like signalling pathways. Their targets were significantly located in cell plasma and organelles, enriched in metabolic processes, transport and cell process pathways as various regulators. Most of these target genes are involved in signal transduction, transcription factor and cell differentiation. For example, it was found that 24 target genes are involved in a wide range of cellular responses including growth, survival, proliferation, cell cycle, DNA repair, apoptosis.

The above insights provide a blueprint for the pathways corresponding to a small fraction of somatic alterations due to BPA exposure. In addition, the cross-talk between various signalling pathways further complicated the pathway-based analysis. Thus, this study may have to shift toward an integrative network-based approach that accounts for more extensive genomic alteration.

## Discussion

This is the first *in vivo* report of miRNA expression in placenta after BPA exposure, which has been examined in a population of pregnant women undergo a therapeutic abortion occurring in the second trimester of pregnancy than has ever been reported.

Therefore, the aim of this study was to identify changes in placenta miRNA expression impacted by gestational exposure to BPA and to gain a comprehensive understanding of developmental changes in the placenta transcriptome; beside, to determine the impact of gestational exposure to an environmentally relevant dose of BPA on fetuses malformations.

Exposure to BPA at concentrations detected in humans has been reported to affect neurological, cardiovascular and metabolic diseases (such as diabetes), and even cancers [2, 9].

Known the unique weakness of pregnant women and their fetuses and the fact that this chemical can be endocrine disruptor, it is important to examine the level of exposure in such population. It is critical to have bio-monitoring data on a large diverse population of pregnant women, since the health concerns of prenatal exposure to BPA on the developing newborn and the physiological and behavioural changes during pregnancy that can affect exposure to and disposition of chemicals in the body [26]. We chose to examine a population of pregnant women undergo a therapeutic abortion occurring in the second trimester of pregnancy, living in a polluted area of southern Italy and we found a correlation between the placenta BPA level, miR-146a over expression in all placenta from women bearing fetal malformations.

Here, we found that miR-146 expression was increased in placenta from pregnant women, living in a high polluted area, compared with women with a healthy pregnancy; miR-146a expression in patients was positively associated with the BPA level measured in placenta from the same patient group. We have already analyzed (data not shown), miR-146a expression in placenta from pregnant women, living in a non-polluted area, subjected to therapeutic abortion occurring in the second trimester of pregnancy, since the fetuses had malformations. In placenta from such women, miR-146 expression values resembled the level detected in women with a healthy pregnancy. On the other hand, pregnant women living in a polluted area with normal fetuses showed placental miR-146a expression values similar to pregnant women living in a polluted area with malformed fetuses (data not shown).

Environmental chemicals, such as bisphenol. A, can have an epigenetic effects on DNA methylation, histone modification, and expression of non-coding RNAs (including microRNAs) of (BPA). Such finding has expanded our understanding of the etiology of human complex diseases such as cancers and diabetes. Epigenetics is an important mechanism in the capability of environmental chemicals to influence health and disease, and BPA, is epigenetically toxic. Several data from in vitro and *in vivo* models have established that epigenetic modifications caused by *in utero* exposure to environmental toxicants can induce modifications in gene expression that may persist throughout life.

Male offspring rats exposed to DBP/BBP/MBP show phenotypic alterations observed in during the perinatal period which had noteworthy similarities with common human reproductive disorders, including cryptorchidism, hypospadias and low sperm counts [24]. Mixtures and bisphenol A display additive interactions showing antiandrogenic activities [25].

Exposure to low doses of bisphenol A (BPA) during *in utero* and neonatal stage may cause DNA hypermethylation/hypomethylation at CpG islands near gene promoter regions, histone modifications (acetylation, methylation, phosphorylation, ubiquitination, sumoylation and ADP ribosylation), and altered expression of non-coding RNAs, as microRNAs. These epigenetic changes can bring to up/down alterations in gene expression persist throughout all the life. In turn, such permanent changes will result in adverse health effects such as neural and immune disorders, infertility, and late-onset complex diseases (cancers and diabetes). It has been reported that the transient exposure to BPA of gestating female rats produce differential DNA methylation to the F3 generation.

It has been shown that BPA exposure of human placental cell lines alter miRNA expression levels, and in particular, miR-146a was strongly induced. This resulted in both slower proliferation rate and higher sensitivity to the DNA damaging agent bleomycin [27]. Beside, mouse sertoli cell line TM4 exposed to BPA for 24 h was reported to have two-fold up or down-regulated 37 miRNAs [28].

The discovery that gestational BPA exposure has an effect to increase the expression of most miRNAs within placenta suggests that BPA may impact the processing machinery of miRNAs. Previously, has been demonstrated hormonal regulation of miRNA biogenesis enzymes by steroids, such as estradiol (68). BPA could affect the miRNA biosynthetic pathway by modulating the protein expression or the protein activity of multiple components: beside these particular miRNA genes may have estrogens’ response elements within their promoters, potential target for BPA.

The chance that mir-146a might modulate mRNA levels and subsequent toxicity by BPA has not been explored yet. MiRanda and TargetScan identified 123 conserved genes that are potential targets of the miRNA evaluated in our study.

Using the KEGG pathway database, we identified several pathways that are involved in immune/inflammatory, cancer and neural diseases (Table 3). Figure 3 shows a summary of annotations and analysis of pathways for target genes of miR-146a.

**Table 3:** Genes and genomes pathway analysis of miR-146a target genes

Pathway	Gene Symbol	Gene Name
Apoptosis	IRAK1	interleukin-1 receptor-associated kinase 1,
Cancer	APPL1	adaptor protein, phosphotyrosine interaction, PH domain and leucine zipper containing 1;
	SLC22A3	solute carrier family 22 (extraneuronal monoamine transporter), member 3;
	ERBB4	v-erb-a erythroblastic leukemia viral oncogene homolog 4 (avian);
	NOTCH1	notch 1;
	SMAD4	SMAD family member 4;
	CSF1R	colony stimulating factor 1 receptor;
	DOT1L	DOT1-like, histone H3 methyltransferase (S. cerevisiae);
	PTGFRN	prostaglandin F2 receptor negative regulator;
	IGF2BP1	insulin-like growth factor 2 mRNA binding protein 1;
	BCL11A	B-cell CLL/lymphoma 11A (zinc finger protein);
	RAD1	RAD1 homolog (S. pombe);
	HIC2	hypermethylated in cancer 2;
	MLL2	myeloid/lymphoid or mixed-lineage leukemia 2;
	EDNRB	endothelin receptor type B;
	NOVA1	neuro-oncological ventral antigen 1;
	ABL2	v-abl Abelson murine leukemia viral oncogene homolog 2
Mapk pathway	TRAF6	TNF receptor-associated factor 6
Cell differentation	NOTCH1	notch 1;
	SRP14	signal recognition particle 14 kDa (homologous Alu RNA binding protein);
	PSMD3	proteasome (prosome, macropain) 26S subunit, non-ATPase, 3;
	GDNF	glial cell derived neurotrophic factor;
	SMAD4	SMAD family member 4;
	SEMA3G	sema domain, immunoglobulin domain (Ig), short basic domain, secreted, (semaphorin) 3G;
	MED1	mediator complex subunit 1;
	LIPA	lipase A, lysosomal acid, cholesterol esterase;
	ROBO1	roundabout, axon guidance receptor, homolog 1 (Drosophila);
Metabolic pathway	SLC22A3	solute carrier family 22 (extraneuronal monoamine transporter), member 3;
	PGP	phosphoglycolate phosphatase;
	LIPA	lipase A, lysosomal acid, cholesterol esterase;
	DLST	dihydrolipoamide S-succinyltransferase (E2 component of 2-oxo-glutarate complex);
	DOT1L	DOT1-like, histone H3 methyltransferase (S. cerevisiae);
	BCL11A	B-cell CLL/lymphoma 11A (zinc finger protein;
	DGKG	diacylglycerol kinase, gamma 90 kDa;
Neural disease	IRAK1	interleukin-1 receptor-associated kinase 1;
	MYT1	myelin transcription factor 1;
	ROBO1	roundabout, axon guidance receptor, homolog 1 (Drosophila);
	LRRTM2	leucine rich repeat transmembrane neuronal 2;
	GRID1	glutamate receptor, ionotropic, delta 1;
	SORT1	sortilin 1;
	BCL11A	B-cell CLL/lymphoma 11A (zinc finger protein);
	SYT1	synaptotagmin I;
	NPAS4	neuronal PAS domain protein 4;
	MLL2	myeloid/lymphoid or mixed-lineage leukemia 2;
	DNAL1	dynein, axonemal, light chain 1;
	EDNRB	endothelin receptor type B;
Binding protein	NOVA1	neuro-oncological ventral antigen 1;
	HNRNPD	heterogeneous nuclear ribonucleoprotein D
Signal trasduction	ABL2	v-abl Abelson murine leukemia viral oncogene homolog 2;
	NUMB	numb homolog (Drosophila);
	CNTFR	ciliary neurotrophic factor receptor;
	SRP14	signal recognition particle 14 kDa (homologous Alu RNA binding protein);
	GDNF	glial cell derived neurotrophic factor;
	SMAD4	SMAD family member 4;
	KCTD15	potassium channel tetramerisation domain containing 15;
	NF2	neurofibromin 2 (merlin);
	KLF4	Kruppel-like factor 4 (gut);
	SCN3B	sodium channel, voltage-gated, type III, beta;
	NLGN1	neuroligin 1;
	RIMS2	regulating synaptic membrane exocytosis 2;
Cardiovascular disease	ABL2	v-abl Abelson murine leukemia viral oncogene homolog 2;
	EIF4G2	eukaryotic translation initiation factor 4 gamma, 2;
	EDNRB	endothelin receptor type B;
Ubiquitin pathway	DCAF12	DDB1 and CUL4 associated factor 12;
	FBXW2	F-box and WD repeat domain containing 2;
	WWP2	WW domain containing E3 ubiquitin protein ligase 2;
	DTL	denticleless homolog (Drosophila);
Trasport protein	SLC10A3	solute carrier family 10 (sodium/bile acid cotransporter family), member 3;
	ERBB4	v-erb-a erythroblastic leukemia viral oncogene homolog 4 (avian);
	EIF4G2	eukaryotic translation initiation factor 4 gamma, 2;
	SH3GL2	SH3-domain GRB2-like 2;
	SLCO3A1	solute carrier organic anion transporter family, member 3A1;
	GOSR1	golgi SNAP receptor complex member 1;
	SLC39A1	solute carrier family 39 (zinc transporter), member 1;
	SORT1	sortilin 1;
	BTG2	BTG family, member 2;
Enzyme	ERBB4	v-erb-a erythroblastic leukemia viral oncogene homolog 4 (avian);
	ADARB1	adenosine deaminase, RNA-specific, B1;
	USP3	ubiquitin specific peptidase 3;
	KDM2B	lysine (K)-specific demethylase 2B;
	SIAH2	seven in absentia homolog 2 (Drosophila);
	CASK	calcium/calmodulin-dependent serine protein kinase (MAGUK family);
	LIPA	lipase A, lysosomal acid, cholesterol esterase;
	CNOT6L	
	TRDMT1	CCR4-NOT transcription complex, subunit 6-like;
	MPPE1	tRNA aspartic acid methyltransferase 1;
	DCP1A	DCP1 decapping enzyme homolog A (S. cerevisiae);
	DLGAP2	discs, large (Drosophila) homolog-associated protein 2;
Kinase protein associated	TRAF6	TNF receptor-associated factor 6;
	PCDH1	protocadherin 1;
Prion disease	NOTCH1	notch 1
Infection disease	APPL1	adaptor protein, phosphotyrosine interaction, PH domain and leucine zipper containing 1;
	PGP	phosphoglycolate phosphatase;
	PSMD3	proteasome (prosome, macropain) 26S subunit, non-ATPase, 3;
	SMAD4	SMAD family member 4;
	TAF9B	TAF9B RNA polymerase II, TATA box binding protein (TBP)-associated factor, 31 kDa;
	CD28	CD28 molecule;
	WASF2	WAS protein family, member 2;
	SRSF7	serine/arginine-rich splicing factor 7;
Trascriptor factor	MAFF	v-maf musculoaponeurotic fibrosarcoma oncogene homolog F (avian);
	CSF1R	colony stimulating factor 1 receptor;
	TAF9B	TAF9B RNA polymerase II, TATA box binding protein (TBP)-associated factor;
	EIF5A2	eukaryotic translation initiation factor 5A2;
	KLF4	Kruppel-like factor 4 (gut);
	SP8	Sp8 transcription factor;
	SOX5	SRY (sex determining region Y)-box 5;
Endocrine system	MED1	mediator complex subunit 1;
	TP53RK	TP53 regulating kinase;
Cellular antigen	CD84	CD84 molecule
Mirna survelliance	PPP2R3A	protein phosphatase 2, regulatory subunit B’, alpha;
	RIMS2	regulating synaptic membrane exocytosis 2;

Although the molecular events underlying the effects of developmental BPA exposure in humans remain unclear, they appear to involve pathways beyond classical Estrogen Receptor signalling. The identification of neural diseases pathway was one among the significant biological functions predicted for miR-146a gene targets. Notably, all of the 12 genes comprising this function, exhibited roles in impaired nervous system functioning such as GRID1, which mediate most of the fast excitatory synaptic transmission in the central nervous system, play key roles in synaptic plasticity and increased risk of developing schizophrenia. ROBO1, implicated in communication disorder as dyslexia and communication disease. SORT1, which is the key contributors to aneurysmal disease. Mir-146a potentially interacts with genes which are involved in cardiovascular diseases too as ABL2, that mediate vascular permeability, and EDNRB gene providing instructions for making a protein called endothelin receptor type B. Endothelins regulate several critical biological processes, including the development and function of blood vessels, Regarding Endocrine system regulation, in particular, hsa-mir-146a has complementary sequence for 3′-UTR region of endocrine system genes as MED1, which has been shown may interact with Androgen receptor, Estrogen receptor alpha, and p53. The main function of the androgen receptor is as a DNA-binding transcription factor that regulates gene expression; however, the androgen receptor has other functions as well. Androgen regulated genes are critical for the development and maintenance of the male sexual phenotype. The estrogen receptor (ESR) is a ligand-activated transcription factor composed of several domains important for hormone binding, DNA binding, and activation of transcription. TP53RK gene, encoding TP53-regulating kinase is a miR-146a target. Distinct stresses, including ionising radiation, virus infection and metabolic stress in the form of altered AMP/ATP ratios, can induce p53 phosphorylation.

Nishizawa et al. [29, 30] found that placental gene expression after BPA exposure were studied in experiments that focused on retinoic acid (RA) receptors (RAR and RXR) and aryl hydrocarbon receptors (AhR). RA acts by binding to RA receptors RAR and RXR, which dimerize to form transcription factors. In a series of papers, Nishizawa et al. advanced the hypothesis that BPA can activate AhR expression in embryos, thus subsequently altering RA action during embryogenesis. BPA was administered orally during organogenesis to mice and embryos were collected for the analysis of mRNA expression by RT-PCR. In further support of AhR activation, expression of two other genes in this pathway was also increased. CYP1A1 and GST (two enzymes regulated by AhR) mRNA and protein expression were increased at the higher doses of BPA and with E2.

The ability of BPA to alter miRNA expression in the placenta might reflect a mechanism of BPA toxicity which may bring harmful consequences for the developing fetus. In the placenta, normal miRNA regulation in placental tissue is extremely important due to growth-promoting and the growth-inhibiting roles of human miRNAs.

## Conclusions

In the environment the enhancing presence of BPA point to look for a comprehensive examination of human responses to BPA exposure, including molecular effects. The significance of studying BPA’s consequences on the placenta is accentuated by BPA’s tendency to accumulate in such tissue and the general importance of placenta in fetal development.

In summary, our findings suggest that BPA are able to modify miRNAs expression in humans. In particular, mir-146a is correlated significantly with BPA accumulation in the placenta and may be the candidates to serve as promising biomarkers with sufficient sensitivity and specificity for the diagnosis of BPA contamination.

However, the molecular mechanism through which mir-146a might modulate mRNA levels and subsequent toxicity by BPA has not been fully explored. This research serves as a launching point for future studies.

Molecular mechanisms that underlie the long-lasting effects of BPA and phthalates continue to be elucidated, and they likely involve disruption of epigenetic programming of gene expression during development. It will be important to determine whether epigenetic markers in more accessible tissues correlate with epigenetic markers in target tissues. Many studies strongly imply that exposures to endocrine-disrupting chemicals may have cumulative adverse effects on future generations, and that these effects could be mediated through epigenetic mechanisms.

Our research provides to demonstrate the utility of miRNA expression profiles to improve our understanding of environmental toxicants action and may support prevention, diagnosis, and treatment of exposure-related effects.

## Methods

### Ethics statement

We obtained ethics approval for our study from the ethics committee (also known as an Institutional Review Board) at University of Salerno. All the participants had the capacity to consent and we obtained written informed consent from all participants involved in the study. Beside, ethics approval for our study was obtained from the ethics committee (also known as an Institutional Review Board) at our institution.

### Tissue collection

Eighty (40 patients and 40 controls) pregnant women, were enrolled in a case–control study at the Department of Medicine, of the “University of Salerno”. 40 pregnant women living in a non-polluted area (control group: women with a healthy pregnancy,) and 40 pregnant women living in a polluted area (patient group: pregnant women whose fetuses had malformations) from Salerno’ district (Italy), subjected to therapeutic abortion occurring in the second trimester of pregnancy for fetal malformation. Collection was done from June 2013 to July 2014. Additional maternal and infant data were collected from medical records.

All patients met the inclusion criteria: 18–45 years of age, residence in Salerno’ area in the past 5 years, null-parity/no breastfeeding history, absence of immunologic, hormonal disorders, or chronic diseases, and no occupational exposure to BPA s or pesticides.

Sample selection was based on maternal arrival time and time of placental delivery. Placentas were collected from singleton births from HIV and hepatitis negative mothers over 18 years of age. Whole placentas were placed in Whirlpacs® (Nasco) and stored at 80 °C until processing. Prior to analysis, the chorionic plate and decidua basalis were removed with ceramic scissors, leaving the villous core tissue to be homogenized. Samples were oven dried at 60 °C and stored in 50 mL digestion vessels (Environmental Express) at ambient temperature until required for analysis.

### GC-MS analysis

Placental BPA concentrations were determined by GC-MS utilizing a double blind experiment design. Dried and homogenized placental samples (50 mg) were transferred into 2 mL Eppendorf™ vials and 0.5 mL H2O and 1.0 mL of ethyl acetate (Romil, Cambridge, England) containing the internal standard d16-BPA (deuterated Bisphenol A) (ISOTEC; Aldrich, Taufkirchen, Germany) were added. The vials were placed in a 60 °C ultrasonic water bath and sonicated for 30 min. Then 500 mL of the supernatant were transferred to 2 mL glass reaction vials, and the extracts were evaporated to dryness under a stream of nitrogen. Then 50 mL of the derivatizing reagent N,O-bis(trimethylsilyl)trifluoroacetamide (BSTFA) (Supelco, Sigma, Germany) was added. The vials were sealed with Teflon/silicone cap liners and heated in an oven (70 °C) for 2 h. The 2 mL glass vials were removed and 50 mL ethyl acetate were added. Samples of 1 mL from this solution were injected into the GC-MS system (GC-2010 Plus gas chromatograph coupled to a 2010 Plus single quadrupole mass spectrometer; Shimadzu Corp., Kyoto, Japan). Chromatographic separation was achieved with a 60m_ 0.25mmZB-5 ms fused silica capillary column with 0.25 mm film thickness from Phenomenex (Torrance, CA, USA) with helium as carrier gas. The initial oven temperature of 200 °C was held for 3 min and then raised at 20 °C/min to 300 °C. The gas flow was set to achieve a constant linear velocity of 40 cm/s and the split flow was set to 1:10. The mass spectrometer was operated in electron impact (70 eV) mode coupled with selected-ion monitoring. All calibration curves displayed excellent linearity (R2 ¼ 0.9998) over the range of BPA concentrations from 1 to 70 ng/mL. Additional control experiments were performed to ensure that BPA was not leached from the GC-MS itself as well as storage containers and all the tubes and vessels used to collect tissue from patients.

### Statistics

Correlations between placental BPA concentrations and miR-146a were analyzed with a Spearman’s Rank Correlation. Alpha (a) was equal to 0.05.

### RNA isolation

Placenta was collected during the second trimester of pregnancy from all women enrolled. Total RNA was extracted using the TRizol reagent (Invitrogen Carlsbad, CA).

### MicroRNA microarray

Placental microRNA profiles were generated using Human miRNA Microarray kit, 8 × 60 K (based on miRBase release 16.0, Agilent Technologies). The miRNA Complete Labeling and Hyb Kit (Agilent Technologies) was used to label and hybridize 100 ng of total RNA, according to the manufacturer’s instructions. Briefly, RNA samples were dephosphorylated and labeled with cyanine 3-pCp by T4 RNA ligase for 2 h at 16 °C. RNA samples were hybridized to the miRNA microarrays for 20 h at 55° in a hybridization oven at 20 rpm. After washing, the slides were scanned by an Agilent Microarray Scanner (Agilent Technologies). The raw intensity of the array was scanned and extracted by BeadScan, and the data were corrected by background subtraction in the Genome Studio module.

### MicroRNA microarray data analysis

Expression data were extracted from the scanned images using Feature Extraction software, version 10.7 (Agilent Technologies) and analyzed with the R statistical environment (version 2.15.1). The background was adjusted by subtracting the median background values from the median expression values obtained by the Feature Extraction software, followed by log base 2 transformations. The data were quartile normalized by the GeneSpring Gx 12.6.1 software. To calculate sample correlation, a Pearson uncentred correlation was performed with the R package. miRNAs expression levels were considered statistically significant if the difference between the patients and control groups was at least 2.5 fold (*P*-value < 0.05 and percentage of false prediction (pfp) </= 5 %). To obtain a single expression value for each probe set, the median expression value was calculated for multiple probe sets corresponding to a unique miRNA.

### Real-time qPC for miRNA quantification

To quantify miRNA expression levels, microRNA assay-based quantitative RT-PCR (qRT-PCR) was performed for the mature miRNAs, using miScript PCR Starter Kit (Quiagen). Real-time PCR was performed using a 7500 Fast Real-Time PCR system (Applied Biosystems, Foster City, CA, USA), following the ∆-∆Ct method. RNU6B was utilized for an endogenous reference to standardize miRNA expression levels. All RT reactions were run in triplicate. All the data were calibrated by the universal reference data. All results shown are mean +/− SD of at least three separate experiments, measuring each parameter by triplicate (n = 3). Statistical significant differences were tested by one way analysis of variance (ANOVA), and, when the *F* value was significant, by Student-Newman-Keul’ s test. *P* value less than 0.05 (*) was considered statistically significant.

### Target prediction and pathway mining

We used the miRNA target prediction software miRanda (www.microrna.org/) and target scan (www.targetscan.org/) to predict the target genes of miR-146a total of 54 target conserved genes were annotated.

Predicted targets of miR-146a were classified into functional pathways using the Kyoto Encyclopedia of Genes and Genomes, KEGG (KEGG; www.genome.jp/kegg/). A total of 54 KEGG pathways were annotated for miR-146a.

## Abbreviations

BPA: Bisphenol A
miRNAs: MicroRNAs
qPCR: quantitative polymerase chain reaction
KEGG: Kyoto Encyclopedia of Genes and Genomes
AhR: aryl hydrocarbon receptors
